# Ultrasound detection of incidental diffuse parotid disease: A single-center study

**DOI:** 10.1371/journal.pone.0219308

**Published:** 2019-07-03

**Authors:** Do Hun Kim, Dong Wook Kim, Jin Young Park, Yoo Jin Lee, Hye Jung Choo, Tae Kwun Ha, Soo Jin Jung, Ji Sun Park, Sung Ho Moon, Ki Jung Ahn, Hye Jin Baek

**Affiliations:** 1 Department of Otorhinolaryngology-Head and Neck Surgery, Busan Paik Hospital, Inje University College of Medicine, Busan, South Korea; 2 Department of Radiology, Busan Paik Hospital, Inje University College of Medicine, Busan, South Korea; 3 Department of General Surgery, Busan Paik Hospital, Inje University College of Medicine, Busan, South Korea; 4 Department of Pathology, Busan Paik Hospital, Inje University College of Medicine, Busan, South Korea; 5 Department of Nuclear Medicine, Busan Paik Hospital, Inje University College of Medicine, Busan, South Korea; 6 Department of Anesthesiology and Pain Medicine, Busan Paik Hospital, Inje University College of Medicine, Busan, South Korea; 7 Department of Radiation Oncology, Busan Paik Hospital, Inje University College of Medicine, Busan, South Korea; 8 Department of Radiology, Gyeongsang National University Changwon Hospital, Gyeongsang National University School of Medicine, Changwon, South Korea; National Institutes of Health, UNITED STATES

## Abstract

In this study, we compared ultrasound (US) features between normal parotid parenchyma (NPP) and incidental diffuse parotid disease (DPD). From January 2008 to December 2017, 180 patients underwent neck US before parotid surgery at our hospital. From these, 82 were excluded because of the lack of histopathological data concerning the parotid parenchyma or inadequate US images. A single radiologist blinded to the clinicoserological data and histopathological results, retrospectively investigated all US features and categorizations for the parotid glands using a picture archiving and communication system. Retrospective histopathological analysis of the parotid parenchyma was performed by a single pathologist. On the basis of the histopathological analyses, the 98 patients were divided into NPP (n = 70) and DPD (n = 28) groups. Among US features, parenchymal echogenicity and echotexture showed statistically significant differences between the two groups (*p* < 0.0001), whereas the gland size, margin, and vascularity showed no significant differences (*p* > 0.05). The US-based categorization significantly differentiated between NPP and DPD (*p* < 0.0001), and receiver operating characteristic curve analysis revealed that US categorization based on ≥2 abnormal US features showed the best diagnostic performance for detecting DPD. Thus, US can aid in differentiating DPD from NPP.

## Introduction

The major salivary glands, including the parotid, submandibular, and sublingual glands, play an important role in preservation of the oral cavity and dental health [1]. The parotid gland produces saliva in response to eating or the thought or smell of food, whereas the submandibular and sublingual glands produce saliva constantly [1]. Patients with major salivary gland disease may present with symptoms such as dry mouth, dysphagia, duct obstructions, inflammation, and severe dental caries [1]. In clinical practice, salivary gland imaging plays an important role in the visualization of morphology and function, and is used for diagnosis, treatment, and surgical planning [1]. At present, salivary gland imaging includes plain radiography, sialography, ultrasound (US), computed tomography, magnetic resonance imaging, salivary gland scintigraphy, and fluorine-18-labeled flurodeoxyglucose positron emission tomography. US is often used as the initial imaging modality for the assessment of salivary gland disease because of its simplicity, lack of irradiation, and low cost relative to that of other imaging modalities [1, 2]. Salivary gland US can replace scintigraphy or sialography for the diagnosis of primary Sjögren’s syndrome [3–5] and can also be used for prognostic evaluation of patients with this condition [4, 6].

Primary Sjögren’s syndrome is a chronic progressive autoimmune disease of unknown etiology [1]. Sjögren’s syndrome is characterized by lymphocytic infiltration and the obstruction of exocrine glandular tissue, particularly in the lacrimal and salivary glands, thus resulting in xerostomia [1, 6, 7]. Patients with Sjögren’s syndromes should be annually monitored, because they have a 16- to 40-fold increased risk of developing B-cell lymphoma [8, 9]. Thus, US detection of incidental or subclinical Sjögren’s syndrome may help in patient management. To our knowledge, however, no previous studies have investigated the US features of incidental diffuse parotid disease (DPD) or determined the US features that differentiate incidental DPD from the normal parotid parenchyma (NPP). Accordingly, the aims of the present study were to investigate the characteristic US features of incidental DPD and compare the findings with those for NPP.

## Patients and methods

### Patients

This retrospective study was approved by the Busan Paik Hospital institutional review board (IRB 18–0103), and informed patient consent was waived because of the retrospective study design. From January 2008 to December 2017, 180 patients (78 women, 102 men; mean age, 51.7 ± 14.6 years [range, 13–88 years]) underwent neck US including the major salivary glands before parotid surgery at our hospital. Parotid surgery was required for the treatment of known parotid tumors (n = 137) or abscesses (n = 7) or the diagnosis of parotid lesions (n = 36). From the total, 54 patients with insufficient US images or poor-quality US images were excluded, in addition to 28 patients without available histopathological specimens of the parotid gland parenchyma. Eventually, 98 patients (46 women, 52 men; mean age, 51.0 ± 14.6 years [range, 16–75 years]) were included in the study.

### Preoperative ultrasound

For all study patients, preoperative US was performed by two radiologists with 6 and 16 years of experience in performing neck US, respectively. In our hospital, neck US includes the major salivary glands. The procedure was performed with a high-resolution ultrasound scanner (HDI 5000 and iU 22; Phillips Medical Systems, Bothell, WA, USA, and Aplio 400; Toshiba Medical Systems, Tokyo, Japan) equipped with a 5–12-MHz or an 8–15-MHz linear probe. US modalities were randomly chosen for each patient. During color Doppler US, a low pulse repetition frequency (700 Hz), low velocity scale (4.0 or 5.0 cm/s), and gain setting (between 75 and 78) were used.

### Ultrasound imaging analysis

A single radiologist (with 16 years of experience in performing neck US after board certification) retrospectively investigated the US findings of the parotid glands in all patients. This investigator was blinded to the histopathological results and clinicoserological data concerning the parotid gland. The following US features were categorized using a picture archiving and communication system: parenchymal echogenicity (normal, decreased, or increased) and echotexture (fine, coarse, or micronodulation), gland size (normal, increased, or decreased), gland margin (smooth or lobulated), and parenchymal vascularity (normal, decreased, or increased). The adjacent fat and muscle were used as references for determining parenchymal echogenicity. However, the radiologist subjectively decided the size of the parotid gland without concrete criterion. The enrolled patients were subsequently classified into one of four categories according to the number of observed US features: category 1, no abnormal US features; category 2, one abnormal US feature; category 3, two abnormal US features; and category 4, ≥3 abnormal US features.

### Histopathological analysis

Histopathological findings for the parotid gland were retrospectively analyzed by a single pathologist with 18 years of experience in the histopathological analysis of parotid disease after board certification. This investigator was blinded to the US results and serology and assessed residual parotid parenchyma surrounding the mass on the histology slide. Autoimmune parotitis was defined as the progressive loss of glandular cells, with replacement by lymphocytes and formation of germinal centers associated with fibrosis. When there was evidence of inflammation with features that were not compatible with autoimmune parotitis, a diagnosis of nonspecific parotitis was assigned. Diffuse hyperplasia was defined by diffuse hypertrophy and hyperplasia of glandular cells, with retention of the lobular architecture and no definite nodule formation. The parotid gland was considered normal (NPP) when there was no visual evidence of coexisting DPD. When more than 50% lobules were replaced by fat cells, the diagnosis was diffuse fatty change of the parotid gland.

### Statistical analysis

The acquired data were tested for normality of distribution using the Shapiro–Wilk test. In comparisons of US features and categories between the DPD and NPP groups, we used independent t-tests for continuous variables, Pearson’s χ^2^ test for small cell values, and Fisher’s exact test for categorical variables. Continuous variables are expressed as means ± standard deviation. Associations between individual US features and DPD were evaluated for the determination of significant independent predictors of DPD. The Mantel–Haenszel chi-squared test was also used to evaluate the linear association between individual US features and the incidence of DPD. The diagnostic accuracy of the significant US features and US-based categorization for DPD detection were evaluated using receiver operating characteristic (ROC) curve analysis; the area under the ROC curve (AUC) was compared using the method of DeLong et al. [10]. The cut-off value for US-based categorization was determined by maximizing the sum of the sensitivity and specificity.

All statistical analyses were performed with statistical software (SPSS, version 24.0, SPSS; and MedCalc, version 14.10, MedCalc Software). A two-sided *p*-values of <0.05 were considered statistically significant.

## Results

The parotid surgery performed for the 98 patients included excision biopsy (n = 16), unilateral parotidectomy (n = 80), and bilateral parotidectomy (n = 2). The locations of the resected parotid glands were right (n = 51), left (n = 45), and both (n = 2). The postoperative histopathological diagnoses included benign mixed tumor (n = 51), Warthin’s tumor (n = 30), abscess (n = 4), basal cell adenoma (n = 2), myoepithelioma (n = 1), tuberculous lymphadenopathy (n = 1), lipoma (n = 1), oncocytic adenoma (n = 1), mucoepidermoid carcinoma (n = 4), basal cell adenocarcinoma (n = 1), lymphoma (n = 1), and metastasis from squamous cell carcinoma (n = 1). On the basis of the retrospective histopathological analysis of the parotid gland parenchyma, 70 and 28 patients were included in the NPP and DPD groups, respectively. Six (21.4%) patients in the DPD group were histopathologically diagnosed with autoimmune parotitis; the remaining 22 were diagnosed with nonspecific parotitis. Among the 22 patients with nonspecific parotitis, three exhibited concomitant abscesses and one exhibited tuberculous lymphadenopathy on the ipsilateral side. Diffuse hyperplasia cases were not observed in any patients.

**Fig 1:**
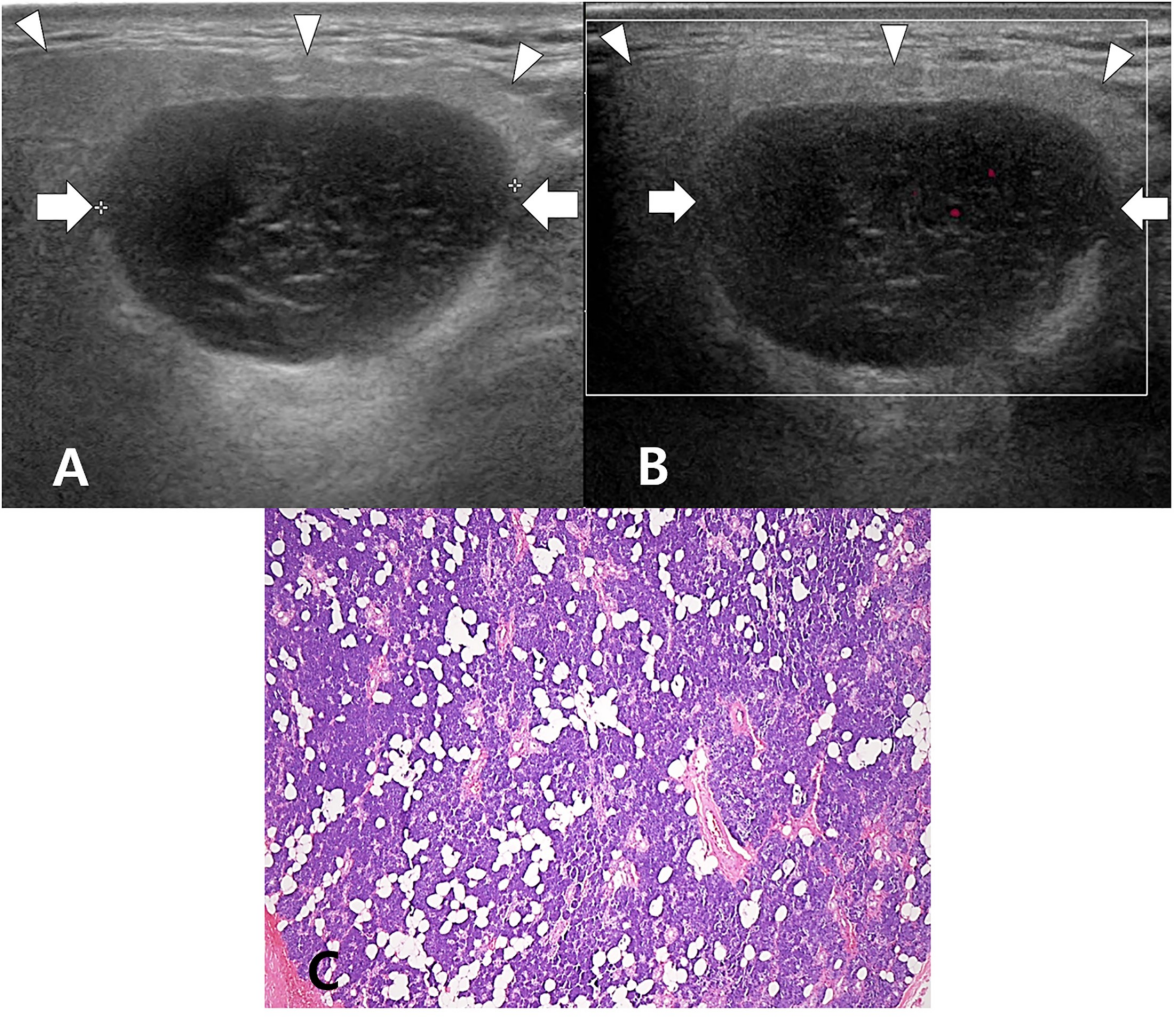
A 45-year-old woman with category 1 on US and normal parotid parenchyma in histopathology. On the longitudinal gray-scale sonogram (A), a Warthin’s tumor (arrows) in the right parotid gland is observed, and the right parotid gland (arrowheads) shows normal parenchymal echogenicity, fine parenchymal echotexture, normal gland size, and a smooth margin. On the longitudinal color Doppler sonogram (B), normal parenchymal vascularity is observed. In the histology slide (C), the right parotid gland shows a normal parenchyma with serous acini and no inflammatory cells (hematoxylin and eosin stain, ×100).

**Fig 2:**
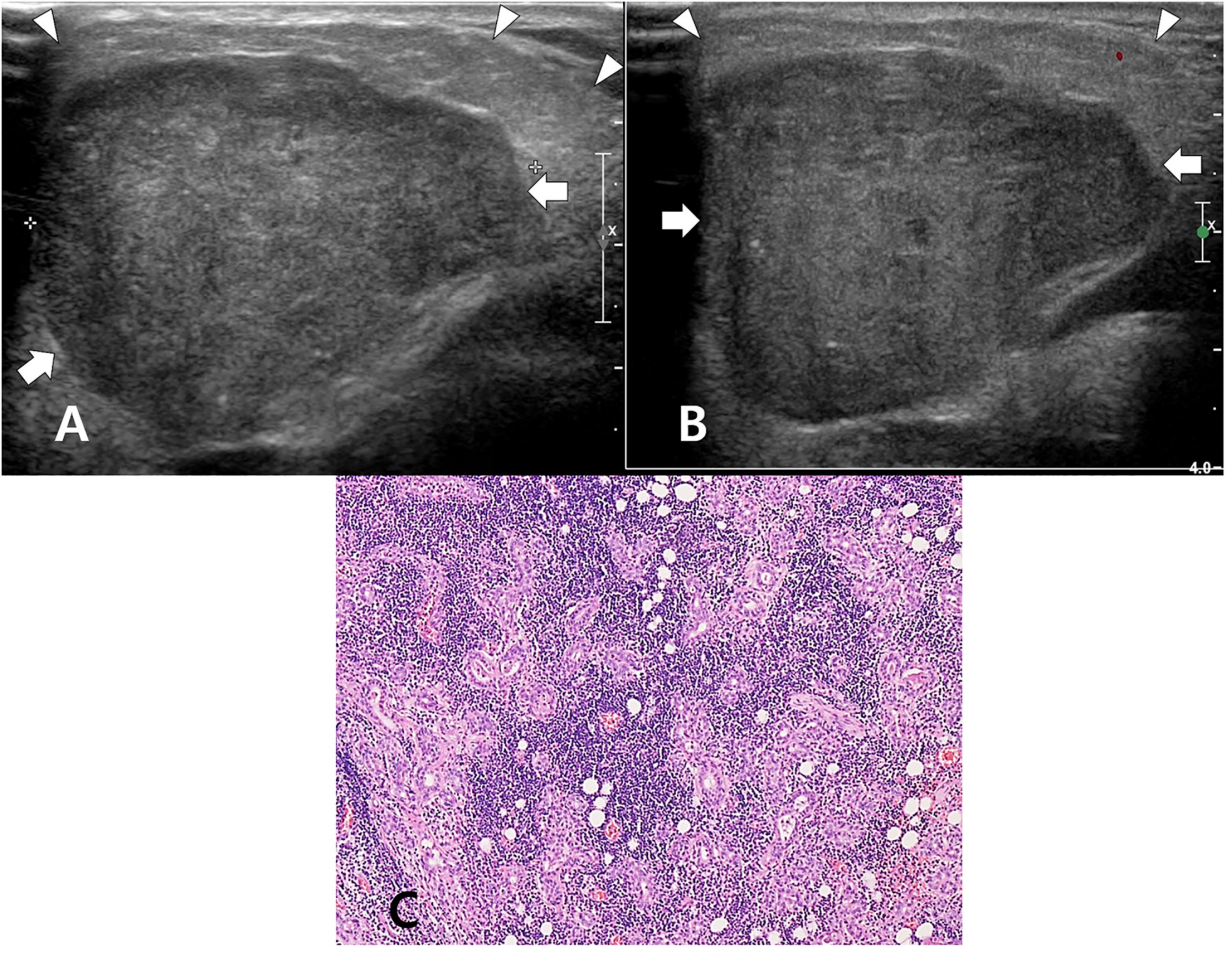
A 55-year-old man with category 3 on US and autoimmune parotitis in histopathology. On the longitudinal gray-scale sonogram (A), a benign mixed tumor (arrows) in the right parotid gland is observed, and the right parotid gland (arrowheads) shows decreased parenchymal echogenicity, coarse parenchymal echotexture, normal gland size, and a smooth margin. On the longitudinal color Doppler sonogram (B), normal parenchymal vascularity is observed. In the histology slide (C), marked loss of glandular acini and dilated ducts surrounded by dense lymphoid cells are noted (hematoxylin and eosin stain, ×100).

The US features for the NPP (Fig 1) and DPD (Fig 2) groups are compared in Table 1 (S1 Table). Among the individual US features, the parenchymal echogenicity and echotexture showed statistically significant differences between the two groups (*p* < 0.0001), whereas gland size, gland margin, and parenchymal vascularity showed no significant differences (*p* > 0.05). US-based categorization exhibited a statistically significant difference between the two groups (*p* < 0.0001).

**Table 1:** Frequency analysis of ultrasound features of the normal parotid parenchyma and diffuse parotid disease in 98 patients.

US features	Normal parotid parenchyma (n = 70)	Diffuse parotid disease (n = 28)	P value
Echogenicity			<0.0001
normal	70 (71.4%)	18 (18.4%)	
decreased	0	10 (10.2%)	
increased	0	0	
Echotexture			<0.0001
fine	56 (57.1%)	2 (2%)	
coarse	14 (14.3%)	25 (25.5%)	
micronodulation	0	1 (1%)	
Gland size			0.08
normal	70 (71.4%)	26 (26.5%)	
decreased	0	2 (2%)	
increased	0	0	
Glandular margin			0.096
smooth	64 (65.3%)	22 (22.4%)	
lobulated	6 (6.1%)	6 (6.1%)	
Vascularity			0.286
normal	70 (71.4%)	27 (27.6%)	
decreased	0	0	
increased	0	1 (1%)	
US categorization			<0.0001
Category 1	55 (56.1%)	1 (1%)	
Category 2	10 (10.2%)	11 (11.2%)	
Category 3	5 (5.1%)	15 (15.3%)	
Category 4	0	1 (1%)	

Note.—Data presented in parentheses are percentage of each item. US, ultrasound; DPD, diffuse parotid disease.

Histopathology revealed diffuse fatty change in 13 (13.3%) patients, only three (23.1%) of whom exhibited DPD. There was no significant relationship between diffuse fatty change and the parenchymal echogenicity (*p* = 0.749) and echotexture (*p* = 0.917), gland size (*p* = 1.000), gland margin (*p* = 0.659), parenchymal vascularity (*p* = 0.133), and the US-based category (*p* = 0.933).

**Fig 3:**
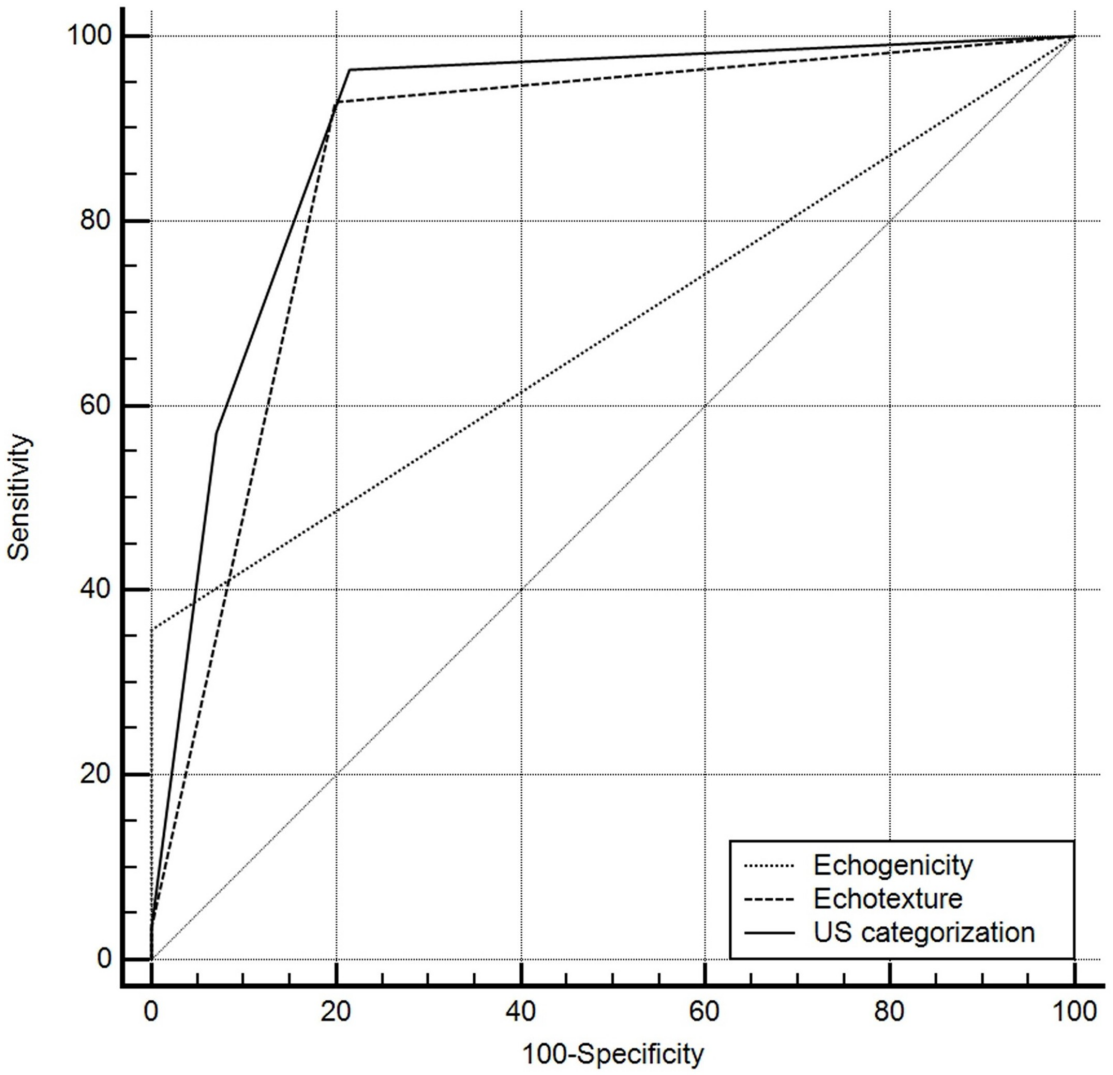
Comparison of diagnostic performance of each independent ultrasound (US) predictor for detecting diffuse parotid disease (DPD). For the US categorization, category 3 (≥2 abnormal US features) was used as a cut-off value. The diagonal line represents 50% of the area under the receiver operating characteristic (ROC) curve and refers to a hypothetical marker that has no discriminatory power for differentiating DPD from normal parotid parenchyma.

ROC curve analysis revealed that category 3 (≥2 abnormal US features) showed the highest diagnostic performance in terms of DPD detection (*p* = 0.0035) (Fig 3). The diagnostic performance of each independent predictor of DPD is shown in Table 2.

**Table 2:** Diagnostic performance of individual ultrasound features and ultrasound-based categorization for the detection of diffuse parotid disease in 98 patients.

US features	Az value*	Sensitivity	Specificity	PPV	NPV	Accuracy	P value
Echogenicity	0.679 (0.577, 0.769)	35.7%	100%	100%	79.5%	81.6%	0.0001
Echotexture	0.868 (0.784, 0.928)	92.9%	80%	65%	96.6%	83.7%	<0.0001
US categorization (cutoff: category 3)	0.903 (0.827, 0.954)	96.4%	78.6%	64.3%	98.2%	83.7%	<0.0001

Note.—Az means the largest area under the ROC curve.

*Numbers in parentheses are 95% confidence intervals.

NPV, negative predictive value; PPV, positive predictive value; and US, ultrasound.

## Discussion

The existing literature demonstrates that US is helpful for the detection of asymptomatic or subclinical diffuse thyroid disease [11–13], with useful features including the parenchymal echogenicity and echotexture, gland margin and size, and parenchymal vascularity [11–13]. In the present study, we hypothesized that the same US features could be used for the detection of asymptomatic or subclinical DPD and its differentiation from NPP.

Accordingly, we investigated five US features and found that only the parenchymal echogenicity and echotexture were helpful for the detection of DPD at a statistically significant level. This result differs from those of previous thyroid US studies, which found significance for all analyzed US features [11–13]. The reason for this discrepancy is unclear, although difficulty in the evaluation of the gland margin, gland size, and parenchymal vascularity in the present study could be a reason. Parotid US differs from thyroid US in that there is no specific criterion for measurement of the gland size. Moreover, the normal parotid gland exhibits low parenchymal vascularity on color Doppler US.

With regard to the US-based categories, we found that category 3 (≥2 abnormal US features) exhibited the highest diagnostic accuracy in terms of DPD detection. This indicates that the diagnostic accuracy increases with an increase in the number of abnormal US features, similar to the findings in previous thyroid US studies [11–13]. However, we did not investigate the specific type of DPD, such as Sjögren’s syndrome or other autoimmune parotitis. In clinical practice, Sjögren’s syndrome is the most important type of DPD, because it is the main cause of xerostomia and increases the risk of B-cell lymphoma [1, 6, 7]. Therefore, further US studies investigating the specific DPD are required.

We also investigated diffuse fatty change in the present study and found that none of the cases exhibited increased parenchymal echogenicity. Furthermore, diffuse fatty change exhibited no significant relationship with individual US features or the US-based category. This could be attributed to the low prevalence of diffuse fatty change (13.3%). Further studies are necessary to clarify this aspect.

This study has several limitations. First, all study patients underwent parotid surgery, which may have resulted in selection bias. Second, the image analysis was retrospective for all patients. Accordingly, a limited number of US images were used for analysis. Third, there was no specific criterion for measurement of the gland size, which was determined by the subjective judgement of the radiologist. Fourth, a single radiologist analyzed all US images. Fifth, the histopathological analysis was retrospective, performed by a single pathologist, and based on limited specimens. Most cases exhibited limited parotid parenchyma on the histology slides, which were prepared to focus on the parotid mass. In addition, many cases of excision biopsy (16.3%; 16/98) were included. Finally, a specific diagnosis of the type of DPD could not be established because of the lack of serological data.

### Conclusion

In conclusion, our findings suggest that US can aid in the detection of asymptomatic or subclinical DPD and its differentiation from NPP, with the diagnostic accuracy being the highest when a cut-off of ≥2 abnormal US features is used.

## Supporting information

S1 Table (XLS)
